# Interplay Between Thyroid Hormone Status and Pulmonary Hypertension in Graves’ Disease: Relevance of the Assessment in Thyrotoxic and Euthyroid Patients

**DOI:** 10.3389/fendo.2021.780397

**Published:** 2022-01-06

**Authors:** Larisse Vieira Mendes Araruna, Daniela Camargo de Oliveira, Mônica Corso Pereira, Arnaldo Moura Neto, Marcos Antonio Tambascia, Denise Engelbrecht Zantut-Wittmann

**Affiliations:** ^1^ Endocrinology Division, Department of Internal Medicine, School of Medical Sciences, University of Campinas, São Paulo, Brazil; ^2^ Cardiology Division, Department of Internal Medicine, School of Medical Sciences, University of Campinas, São Paulo, Brazil; ^3^ Pneumology Division, Department of Internal Medicine, School of Medical Sciences, University of Campinas, São Paulo, Brazil

**Keywords:** Graves’ disease, pulmonary hypertension, hyperthyroidism, autoimmune thyroid disease, thyroid hormone

## Abstract

**Background:**

Graves’ disease (GD) is the most common cause of hyperthyroidism and can cause cardiac changes, such as pulmonary hypertension.

**Methods:**

This is a prospective study in which we obtained demographic, clinical, laboratory data and characteristics of the GD, in addition to investigating cardiorespiratory function, focusing on the detection of pulmonary hypertension. Patients were separated into two groups: thyrotoxicosis and euthyroidism. Ninety patients with GD of both sexes, over 18 years of age, were included. The cardiorespiratory assessment included an echocardiographic evaluation, a questionnaire of specific symptoms, spirometry and a six-minute walk test.

**Results:**

The hyperthyroid group included 42 patients (47.73%) and the euthyroid group 46 patients (52.27%); 78 were women (86.67%). The prevalence of pulmonary hypertension between the hyperthyroidism (48.57%) and the euthyroidism (29.41%) groups was not different. Free thyroxine levels (FT4) (OR 1.266), higher left atrium volume (OR 1.113) and right ventricle diameter were associated with pulmonary hypertension. A direct correlation between FT4 with forced vital capacity (FVC) and forced expiratory volume in the first second (FEV1), as also an inverse correlation between initial oxygen saturation (SpO2) with diagnostic time and drop SpO2 with the ratio between the diastolic velocity E of the mitral flow and the diastolic velocity of the mitral ring (E/e’) were observed in the euthyroid group. An inverse correlation between FT4 levels with walked distance as % of predicted value, and a direct correlation between E/e’ ratio and walked distance as % of predicted value were observed in the hyperthyroid group.

**Conclusion:**

We emphasize the importance of a cardiorespiratory reassessment in GD, even after a long-term control of the thyrotoxic state, as we demonstrate that about 30% of these patients remain with PH and are subject to specific treatment.

## Introduction

Graves’ disease (GD) is the most common cause of hyperthyroidism causing changes in the cardiorespiratory system, such as tachycardia, increased pulse pressure, dyspnea and, not uncommonly, pulmonary hypertension (PH) (1–5).

PH is defined as the mean pulmonary arterial pressure higher than 20 mmHg (6, 7), requiring a high degree of suspicion due to its low prevalence and unspecific signs and symptoms. Classically, it includes dyspnea on exertion, lower limb edema, hepatomegaly due to hepatojugular reflux, and symptoms of low cardiac output such as lipothymia and syncope (4, 8). PH classification includes Pulmonary Arterial Hypertension (PAH) idiopathic, heritable, drug-induced and associated with connective tissue diseases (6). PH has multiple causes, such as left heart diseases, hypoxic chronic lung diseases, and chronic thromboembolism. PH associated with thyroid diseases encompasses systemic manifestations in which the pathophysiological mechanisms involved are unclear (6, 9–13).

Right heart catheterization is the gold standard for PH diagnosis; however, Doppler echocardiography is usually the method for screening. In most cases, PH is diagnosed by means of the requested echocardiogram due to other suspected cardiac changes, and even so adequate follow-up and management are not always performed (8).

A series of cases (14, 15) and prospective studies with a small casuistic (16) demonstrating reversal of changes in PAH with hyperthyroidism treatment, uses echocardiography as a diagnostic and follow-up tool. However, other authors report persistence of high levels of pressure in the pulmonary circulation. In such cases, it is important to exclude other causes and associated conditions, before attributing PH only to thyroid dysfunction (14, 15, 17).

The main objective of this study was to assess GD patients for the detection of PH, in relation to thyroid hormonal status, both in the presence of thyrotoxicosis or in euthyroidism after controlling the disease. The specific objectives are the thorough cardiorespiratory assessment of both groups.

## Materials and Methods

### Study Design

This prospective study which assessed GD attended at the Thyroid Unit of a tertiary Endocrinology Service through a directed interview, demographic, clinical and laboratory data, besides the cardiorespiratory function to detect PH. Patients were separated into 2 groups:

a) In Thyrotoxicosis: GD in overt hyperthyroidism, but under treatment with antithyroid drugs during routine medical intervention, aiming at adequate control of thyroid function;

b) In Euthyroidism: hyperthyroidism due to Graves’ disease controlled for at least 1 year with the use of thionamides or in remission of the disease or in replacement treatment with levothyroxine due to hypothyroidism after radioiodotherapy or thyroidectomy.

The joint investigation with the Pulmonology and Cardiology Services, consisted of a questionnaire to assess respiratory symptoms, spirometry, a pulse oximetry, a six-minute walk test (6MWT) and a transthoracic echocardiogram. Biochemical data were serum TSH (reference values RV 0.41–4.5 mUI/L), free thyroxine (FT4) (RV 0.9–1.7 ng/dl), free triiodothyronine (FT3) (RV 0.22–0.44 ng/dl), anti-thyroglobulin antibodies (TgAb) (RV <115 mUI/L), anti-thyroidperoxidase antibodies (TPOAb) (RV <35 IU/ml), and thyroid stimulating hormone receptor antibody (TRAb) (RV <1.58 IU/ml), all measured by electrochemiluminescence immunoassay. Hyperthyroidism was confirmed by elevated FT4, suppressed TSH and thyroid autoimmunity by elevated TgAb, TPOAb, and TRAb.

Written consent was obtained from the patients and the study was approved by the local Ethics Committee (CAAE 02109412.30000.5404).

### Patients

Ninety GD patients, both sexes, over 18 years old, were included, but 2 of them who did not undergo most assessments, were excluded. Transthoracic echocardiogram was performed in 70 of 88 patients and the respiratory assessment was completed in 79 of 88 patients. Patients with acute sickness or recent previous cardiovascular event, malignant neoplasia or active inflammatory disease, connective tissue diseases, use of amiodarone or iodinated contrast less than 3 months before the study, heart failure (NYHA class III or IV), severe liver disease, advanced kidney disease (stage 4 or 5), under hemodialysis, HIV-positive and hepatitis C infection and other autoimmune diseases were excluded.

### Cardiorespiratory Assessment

The cardiorespiratory assessment included an echocardiography, a questionnaire of specific symptoms, spirometry and a six-minute walk test.

*A) Transthoracic echocardiogram:* performed on a Toshiba Xario echocardiograph, with a 3 MHZ sectoral transducer, with two-dimensional evaluation, M mode, pulsed Doppler, continuous Doppler, tissue Doppler and color Doppler, according to the American Society of Echocardiography (18). Assessment carried out the anatomy of left and right chambers, whether or not with structural heart disease that can lead to PH, estimate of left atrial (LA) volume, right ventricle (RV) diameter, left ventricular ejection fraction by the Simpson method (LVEF), cardiac output (CO) derived by Simpson systolic volume, cardiac index (CI), evaluation of systolic or diastolic dysfunction of the left ventricle, mitral regurgitation, systolic pulmonary artery pressure (SPAP) estimate by velocity of tricuspid regurgitation (if present) and right atrial pressure (RAP) by inferior vena cava observation, mean pulmonary artery pressure (mPAP) (19), the ratio between the diastolic velocity (E) of the mitral flow and the diastolic velocity of the mitral ring (E/e’), pulmonary capillary Wedge pressure (PCWP) (20), pulmonary flow velocity, acceleration and ejection time of pulmonary flow, tricuspid annular plane systolic excursion (TAPSE), presence of pericardial effusion, or paradoxical motion of interventricular septum, pulmonary vascular resistance (PVR) (21) and hyperdynamic state.

The references values were LVEF Simpson >52% in men and >54% in women, SPAP up to 35 mmHg, mPAP up to 20 mmHg, RV diameter up to 27 mm, PCWP of 8–12 mmHg, LA up to 34 ml/m^2^, E/e’ up to 12 and TAPSE >17, CI from 2.8 to 4.2, CO up to 4,000 ml/s. The echocardiographic assessment was performed by the same cardiologist expert in echocardiography (DCO).

*B) Degree of dyspnea: Medical Research Council scale (MRC):* Dyspnea on exertion, orthopnea, paroxysmal nocturnal dyspnea, chest pain or oppression, any previous episode of syncope, cough, expectoration, wheezing. The dyspnea on exertion followed the modified MRC (**Chart 1**); MRC 1 and 2: asymptomatic and MRC 3 to 5: symptomatic patients.

Chart 1 **Dyspnea scale of the medical research council (22).**

* 1—Not troubled by breathless except on strenuous exercise*

* 2—Short of breath when hurrying on a level or when walking up a slight hill*

* 3—Walks slower than most people on the level, stops after a mile or so, or stops after 15 min walking at own pace*

* 4—Stops for breath after walking 100 yards, or after a few minutes on level ground*

* 5—Too breathless to leave the house, or breathless when dressing/undressing*

*C) Spirometry:* All patients performed spirometry (without and with bronchodilator) and had Oxygen Saturation (SpO2) measurement in room air. Spirometry was performed with the EasyOne Wordspirometer^®^ (23), and oximetry, with a Nonin^®^ pulse oximeter. We evaluated the forced expiratory volume in the first second (FEV1) in liters (L) and percentage of predicted value (%), the forced vital capacity (FVC) in liters (L) and percentage of predicted value (%), and FEV1/FVC ratio. We consider FVC and FEV1 >80% of the predicted as normal.

*D) Six-minute walk test:* The distance walked and the drop in SpO2 were evaluated. We assessed the distance covered in meters and in percentage of the predicted, initial and final oxygen saturation, final–initial delta SpO2, and initial and final heart rate. We consider the drop in SpO2 ≥4 points as severe.

The test was performed inside a hospital corridor on a hard and flat surface, according to international recommendation (24).

### Statistical Analysis

Statistical analysis was performed by means of frequency tables of categorical variables with absolute frequency (n) and percentage (%) values, descriptive statistics of numerical variables with mean, standard deviation, minimum and maximum values and median. Chi-square or Fisher’s exact tests were used to compare categorical variables. Mann–Whitney test and ANOVA for repeated measures were used for numerical variables. A logistic regression analysis assessed factors associated to PH. Spearman’s correlation coefficient was used for correlate numerical variables and Mann–Whitney and Kruskal–Wallis tests, to compare categorical and numerical variables, respectively. The level of significance adopted was 5%.

## Results

### Clinical, Demographic and Laboratory Characteristics of GD Patients

#### Comparative Analysis Regarding Thyroid Hormone Status

The 88 GD patients were divided in 42 hyperthyroid (47.73%) and 46 euthyroid patients (52.27%); 78 were women (86.67%), with a similar frequency of genders between groups. The age and time of diagnosis were lower in the hyperthyroid group than in the euthyroid group. TSH was lower, FT4 and FT3 were higher in the hyperthyroid group compared to the euthyroid group (**Table 1**).

**Table 1:** Comparative analysis between demographic and laboratory characteristics of patients with Graves’ disease according to thyroid hormone status.

Variable		Euthyroidism	Hyperthyroidism	p-value
Sex	Male	6 (13.04%)	6 (14.29%)	0.8653
	Female	40 (86.96%)	36 (85.71%)	
Age (years)		48.18 ± 10.18 (47, 29–72)	42.07 ± 13.38 (40, 19–72)	0.0091
Ophthalmopathy		14 (31.82%)	17 (40.48%)	0.4032
Body surface area (kg/m^2^)		1.74 ± 0.21 (1.72, 1.41–2.25)	1.7 ± 0,17 (1.7, 1.42–2.07)	0.5321
Betablocker use		9 (20.93%)	15 (36.59%)	0.1124
Smoking	Active	5 (10.87%)	7 (17.07%)	0.6349
	Previous	9 (19.57%)	9 (21.95%)	
TSH (mUI/L)		2.43 ±1.29 (2.37, 0.37–5.72)	0.02 ± 0.04 (0.01, 0.01–0.23)	<0.0001
FT4 (ng/dl)		1.27 ± 0.25 (1.25, 0.45–1.74)	3.62 ± 2.53 (2.41, 0.81–7.77)	<0.0001
FT3 (pg/ml)		0.3 ±0.06 (0.29, 0.22-0.49)	1.16 ± 0.96 (0.67, 0.21–3.25)	<0.0001

TSH, thyroid-stimulating hormone; FT4, free thyroxine; FT3, free tri-iodothyronine.

Categorical variables are represented by the absolute value and frequency in percentage. Continuous variables are represented by values of mean ± standard deviation (median, minimum, and maximum).

#### Comparative Analysis for the Presence of Pulmonary Hypertension

Higher FT4 and FT3 were observed in patients with PH (**Table 2**).

**Table 2:** Comparative analysis between the parameters used in the evaluation of demographic and laboratory characteristics of patients with Graves’ disease according to the presence of Pulmonary Hypertension.

Variable		Pulmonary Hypertension		p-value
		Yes	No	
Sex	Male	4 (14.81%)	6 (13.95%)	1.0000
	Female	23 (85.19%)	37 (86.05%)	
Age (years)		48.3 ± 13.75 (49, 25–72)	43.26 ± 10.95 (43, 19.95–72)	0.0943
Ophthalmopathy		12 (44.44%)	11 (26.19%)	0.1165
Tabagism	Active	1 (3.7%)	5 (11.9%)	0.4235
	Previous	8 (29.63%)	8 (19.05%)	
Betablocker use		12 (44.44%)	9 (22.5%)	0.0575
Active GD (methimazole)		18 (69.23%)	20 (47.62%)	0.0811
Aftter ablative therapy (levothyroxine)		5 (18.52%)	12 (28.57%)	0.3443
Diagnosis time (years)		7 ± 4.98 (5, 1–17)	7.88 ± 7.39 (5, 1–31)	0.9598
Body surface area (kg/m^2^)		1.73 ± 0.18 (1.75, 1.41–2.10)	1.71 ± 0.19 (1.42–2.15)	0.6710
TSH (mUI/L)		0.98 ± 1.4 (0.01, 0.01–4.29)	1.45 ± 1.66 (0.92, 0.01–5.72)	0.1243
FT4 (ng/dl)		3.3 ± 2.63 (1.72, 0.98–7.77)	2.09 ± 1.89 (1.42, 0.45–7.77)	0.0296
FT3 (pg/ml)		1.16 ± 1.06 (0.58, 0.28–3.25)	0.6 ± 0.7 (0.31, 0.21–3.25)	0.0036

TSH, thyroid-stimulating hormone; FT4, free thyroxine; FT3, free tri-iodothyronine.

Categorical variables are represented by the absolute value and frequency in percentage. Continuous variables are represented by values of mean ± standard deviation (median, minimum, and maximum).

### Cardiorespiratory Characteristics

#### Comparative Analysis Regarding Thyroid Hormone Status

PH prevalence between the hyperthyroid (48.57%) and the euthyroid (29.41%) groups was not significantly different, as well as SPAP (35 vs 28.5 mmHg). The hyperthyroid group showed lower PCWP than the euthyroid group (10 vs 13 mmHg), similarly to E/e’ ratio (6 vs 8 mmHg) (**Table 3**). No difference in respiratory assessment was observed between the groups with or without PH (data not shown).

**Table 3:** Comparative analysis between the parameters used in the echocardiographic assessment of patients with Graves' disease according to thyroid hormonal status.

Echocardiographic Evaluation	Euthyroidism	Hyperthyroidism	p-value
Pulmonary hypertension	10 (29.41%)	17 (48.57%)	0.1030
Increased left atrium	4 (12.9%)	4 (12.2%)	1.0000
Diastolic dysfunction	9 (2.68%)	6 (16.22%)	0.4189
Mitral insufficiency	11 (28.95%)	16 (43.24%)	0.1972
Hyperdynamic state	2 (5.26%)	6 (15.38%)	0.2626
LVEF (%)	63.78 ± 8.33 (66, 45–79)	60.89 ± 7.62 (60, 42–79)	0.0948
Right ventricle diameter (mm)	22.09 ± 5.58 (22, 12–36)	23.08 ± 6.97 (22 ± 9–47)	0.6472
SPAP (mmHg)	29.65 ± 6.8 (28.5, 18–43)	35.06 ± 11.45 (35, 18–58)	0.0600
mPAP (mmHg)	14.92 ± 8.9 (12, 2–34)	18.97 ± 10.15 (19.5, 2–44)	0.1073
PCWP (mmHg)	14.22 ± 5.75 (13, 7–28)	10.85 ± 2.89 (10, 7–20)	0.0139
PVR (woods)	1.33 ± 0.42 (1.4, 0.3–2)	1.37 ± 0.41 (1.32, 0.4–2.3)	0.9777
Left atrium volume (ml/m^2^)	24.1 ± 9.05 (24, 11–50)	24.53 ± 8.42 (24, 11–45)	0.8269
TAPSE	23.22 ± 4.27 (22, 17–30)	21.11 ± 3.1 (21, 14–25)	0.4219
E/e’	8.9 ± 4.29 (8, 4–23)	6.83± 1.98 (6, 4–13)	0.0415
Cardiac output (L)	4,893.43 ± 1,708.39 (4,961, 2,494–9,245)	5047.74 ± 1674.32 (4832, 2870–9398)	0.8243
Cardiac index	2.82 ± 0.90 (2.86, 1.37–4.55)	3.01 ± 1.04 (2.99, 1.69–5.46)	0.5107

LVEF, left ventricular ejection fraction by the Simpson method; SPAP, systolic pulmonary artery pressure; mPAP, mean pulmonary artery pressure; PCWP, pulmonary capillary Wedge pressure; PVR, pulmonary vascular resistance; TAPSE, tricuspid annular plane systolic excursion; E/e’, ratio between the diastolic velocity E of the mitral flow and the diastolic velocity of the mitral ring.

Categorical variables are represented by the absolute value and frequency in percentage. Continuous variables are represented by values of mean ± standard deviation (median, minimum, and maximum).

#### Comparative Analysis for the Presence of Pulmonary Hypertension

LA, PVR, mPAP, and presence of diastolic dysfunction were higher, and LVEF was lower in PH patients (**Table 4**). No difference in respiratory assessment was observed between euthyroidism and hyperthyroidism groups. (Data not shown).

**Table 4:** Comparative analysis between the parameters used in the echocardiographic evaluation of patients with Graves’ disease according to the presence of Pulmonary Hypertension.

Echocardiographic Evaluation	Pulmonary Hypertension		p-value
	Yes	No	
Reduced ejection fraction	5 (18.52%)	6 (13.95%)	0.7386
Increased RV	6 (22.22%)	3 (7.32%)	0.1402
Increased LA	7 (30.43%)	1 (2.78%)	0.0042
Diastolic dysfunction	9 (33.33%)	4 (9.3%)	0.0118
Mitral insufficiency	11 (40.74%)	16 (37.21%)	0.7676
LVEF (%)	59.88 ± 6.31 (59.5, 48–74)	64.14 ± 7.76 (65, 45–79)	0.0222
Right ventricle diameter (mm)	24.7 ± 7.92 (23, 14–47)	21.05 ±4.84 (21, 9–30)	0.1105
mPAP (mmHg)	21.65 ± 10.81 (23, 4–44)	14.34 ± 7.75 (15.5, 2–30)	0.0087
PCWP (mmHg)	13.68 ± 5.98 (12, 7–28)	11.2 ± 2.97 (10, 7–21)	0.2968
PVR (mmHg)	1.45 ± 0.5 (1.5, 0.3–2.3)	1.27 ± 0.32 (1.26, 0.4–2)	0.0149
Left atrium volume (ml/m^2^)	28.68 ± 8.86 (29, 13–45)	21.37 ± 7.88 (21, 11–50)	0.0025
TAPSE	22.75 ± 4.59 (23, 14–30)	21.4 ± 2.46 (21.5, 17–26)	0.3018
E/e’	8.86 ± 4.63 (8, 4–23)	6.85 ± 1.96 (6, 4–13)	0.1515
Cardiac output (L)	4,748.69 ± 1,699.99 (4,791.5, 2,924–9,398)	5,050.09 ± 1,571.83 (5,050, 2,494–9,245)	0.3249
Cardiac index	2.73 ± 0.99 (2.49, 1.39–5.46)	3.01 ± 0.89 (2.99, 1.37–5.12)	0.1472

RV, right ventricle diameter; LA, left atrium volume; LVEF, left ventricular ejection fraction by the Simpson method; mPAP, mean pulmonary artery pressure; PCWP, pulmonary capillary Wedge pressure; PVR, pulmonary vascular resistance; TAPSE, tricuspid annular plane systolic excursion; E/e’, ratio between the diastolic velocity E of the mitral flow and the diastolic velocity of the mitral ring.

Categorical variables are represented by the absolute value and frequency in percentage. Continuous variables are represented by values of mean ± standard deviation (median, minimum, and maximum).

### Logistic Regression Analysis

A univariate analysis for factors associated with PH revealed association with FT4; for each unit of increase in FT4, a 27% greater chance of PH (OR 1.266), with higher left atrium volumes (OR 1.113) and right ventricle diameters (OR 1.103). The presence of diastolic dysfunction increased the chances of PH by 4.8 times (OR 4.875). A multivariate analysis identified increased LA and RV as risk factors for PH (OR 1.217 and 1.251, respectively) (**Table 5**).

**Table 5:** Demographic, laboratory factors of cardiorespiratory assessment associated with pulmonary hypertension in patients with Graves' disease.

Variable	OR	IC 95%	p-value
**Univariate Regression Analysis**			
FT4 (ng/dL)	1.266	1.013–1.583	0.0382
Right ventricle diameter (mm)	1.103	1.008–1.208	0.0328
Left atrium volume (ml/m^2^)	1.113	1.031–1.200	0.0058
Diastolic dysfunction Yes × No	4.875	1.324–17.949	0.0172
Asymptomatic × Symptomatic MRC	1.146	0.248–5.296	0.8616
**Multivariate Regression Analysis**			
Volume left atrium (ml/m^2^)	1.217	1.050–1.410	0.0040
Right ventricle (mm)	1.251	1.027–1.523	0.0155

TSH, thyroid-stimulating hormone; FT4, free thyroxine; FT3, free tri-iodothyronin; FVC, forced vital capacity; FEV1, forced expiratory volume in the first second; Drop SpO2, final–initial SpO2; Asymptomatic MRC, Dyspnea scale of the Medical Research Council between 1 and 2; Symptomatic MRC, Dyspnea scale of the Medical Research Council between 3 and 5.

The regression analysis model included the variables: age, TSH, FT3, predicted distance, predicted distance (%), FVC (L), FVC (%), FEV1 (L), FEV1 (%), drop SpO2, diagnosis time, and thyroid hormone status. Only the variables that proved to be significant were included in **Table 5**.

### Spearman Correlation

Euthyroid presented a direct correlation between FT4 with FVC and FEV1, an inverse correlation between initial SpO2 with diagnostic time and drop SpO2 with E/e’.

Hyperthyroid presented an inverse correlation between FT4 with walked distance as % of predicted value, and a direct correlation between E/e’ ratio and walked distance as % of predicted value in the six-minute walking test. A direct correlation was found between FT4 and FT3 with the final heart rate, as also FT4 and initial heart rate (**Table 6**).

**Table 6:** Comparative analysis between the parameters used in the respiratory assessment of patients with Graves’ disease according to the presence of Pulmonary Hypertension.

	Euthyroidism				Hyperthyroidism				
	Predicted Distance	SpO2 initial	Drop SpO2	FEV1	Predicted Distance	Predicted Distance %	Final Heart Rate	Initial Heart Rate	
SPAP (mmHg)				−0.3800					ρ
				0.0461					p-value
E/e’	−0.5755		−0.4019		−0.5637	0.4463			ρ
	0.0011		0.0418		0.0015	0.0196			p-value
Diagnosis time (years)		−0.3996							ρ
		0.0106							p-value
FT3 (pg/ml)					0.4729		0.4870		ρ
					0.0110		0.0086		p-value
FT4 (ng/dl)				−0.3779	0.3581	−0.4166	0.4231	0.4113	ρ
				0.0211	0.0376	0.0177	0.0081	0.0103	p-value

ρ, Spearman Correlation; SPAP, systolic pulmonary artery pressure; E/e’, ratio between the diastolic velocity E of the mitral flow and the diastolic velocity of the mitral ring; FT3, free tri-iodothyronine; FT4, free thyroxine; Drop O2, final–initial SpO2; FEV1, forced expiratory volume in the first second.

We emphasize that smoking, the use of drugs, body surface area, and other variables investigated did not determine an interference in the results.

## Discussion

The increase in serum thyroxine and triiodothyronine in GD leads to typical cardiorespiratory changes and can develop PH (25, 26).

In the present study, the prevalence of PH was similarly high both in thyrotoxic (48.57%) and in euthyroid patients (29.41%). This finding suggests persistent vascular injury in GD (4), even after the control of hyperthyroidism for at least 1 year. In this sense, we emphasize that FT4 was a factor associated with PH in GD, however, the time in hyperthyroidism was not evident as such in these patients.

PH is present in 35 to 65% of GD hyperthyroid patients (7). The other authors who included patients with Graves’ hyperthyroidism and toxic multinodular goiter found 43% of PH (10). A prospective study evaluating 75 hyperthyroid patients found 47% with SPAP above 35 mmHg on Doppler echocardiography (11). In this study, the prevalence of PH found in GD was high and similar to the literature. This finding emphasizes the importance of cardiorespiratory assessment, since the PH, especially if associated with clinical symptoms, may have an impact on the prognosis and may indicate the need for confirming the diagnosis and even establish a specific treatment. Most authors evaluated the patients during the thyrotoxic state (27), whereas we demonstrated the relevance of the cardiorespiratory assessment, not only in the presence of thyrotoxicosis, but even after the control of hyperthyroidism, in search of persistent injury.

It is noteworthy that SPAP values in GD were similar, whether in thyrotoxicosis or in long-term euthyroidism, corroborating that these patients may present irreversible lesion, with pulmonary artery fibrosis, despite the control thyroid hormone.

The PH pathophysiology associated with hyperthyroidism and GD is not completely understood (12). It is postulated that the excess of thyroid hormones would cause changes in cardiovascular dynamics, with increased heart rate, contraction force, venous return and, consequently, global cardiac output (7). Mechanisms such as increased degradation of vasodilating substances in the pulmonary arterial bed are also implicated, leading to an imbalance between vasoconstriction and vasodilation (28). Endothelial dysfunction occurs, which leads to impairment in the production of vasodilators, such as nitric oxide and prostacyclin, and increased expression of vasoconstrictors and mitogens, such as endothelin-1 (29). Therefore, the finding that FT4 was associated with PH suggests that, in addition to the effects of thyroid hormones on systemic circulation, we can postulate pulmonary vascular effects of hyper-reactivity, vasoconstriction, remodeling, and thrombosis *in situ* (30, 31).

Also, it was reported that patients diagnosed with PH symptoms have a higher frequency of thyroid changes, either hypo or hyperthyroidism, when compared to the general population (12). Autoimmune mechanism is a plausible possibility, with an association between antithyroid antibodies and PH patients (30–33), a fact that would justify, in part, the high prevalence of PH found in patients with GD even after hyperthyroidism control.

Diastolic dysfunction and also the larger volume of the LA and the larger diameter of the RV were shown to be predictive factors of PH in GD. We can suggest that the greater volume of the left atrium is justified in predicting PH, due to the overload induced by diastolic dysfunction, which may be a consequence of PH (34). As for the larger size of RV, it may be related to pressure overload of the pulmonary arterial bed. Such changes would be due to the present or previous thyrotoxic state, which would lead to persistent lesions, or even, could be influenced by the thyroid autoimmunity process (13, 29).

When comparing patients regarding thyroid hormone status, we found, in this study, that those with PH were related to LA and RV increase, but markers of left ventricular diastolic dysfunction, such as elevated values of PCWP and the E/e’ ratio, were associated with euthyroid patients and not with GD in a thyrotoxic state. This find would indicate that, in thyrotoxic state, PH is associated with a precapillary component, and those euthyroid patients may have higher post capillary component.

Similarly, regarding the presence of PH, we found that higher concentrations of both thyroid hormones were associated with PH, which may be justified by the high turnover and overload to the cardiopulmonary system, which originated from the hypermetabolism resulting from excess of circulating thyroid hormones (35).

The echocardiographic evaluation showed that the ejection fraction was lower in the PH group compared to patients who did not have PH, indicating impairment in systemic circulation. In addition, the prevalence of diastolic dysfunction and increased LA was higher in GD patients with PH, as well as PVR and mPAP, causing dysfunction in the systemic circulation, findings that corroborate the damage caused by thyrotoxicosis on the cardiopulmonary system. Such findings warn of the negative impact on cardiac functioning in terms of adequate and effective systemic blood pumping.

Although cardiac catheterization is considered the gold standard method for the PH diagnosis (36), on echocardiography, SPAP can be indirectly estimated by the reflux velocity through the tricuspid valve, thus estimating the gradient between the ventricle and the right atrium, to which is added the estimated pressure of the right atrium. This methodology is widely recognized, has a good correlation with results obtained by cardiac catheterization and, although there is some limitation in the method regarding specificity and sensitivity, it is a non-invasive method, available, of lower cost and without risk to the patient (8). Additionally, mPAP assessed by pulmonary regurgitation, can be considered as a predictive parameter of pulmonary hypertension, with a strong correlation between mPAP values ≥25 mmHg and the increase in SPAP. The evaluation of pulmonary regurgitation is easy to perform, thus, it can be inferred that PH exists when evidencing an increase in mPAP (37). Therefore, we can consider our data as robust from echocardiographic assessment, highlighting that it was performed by a cardiologist expert in the area.

The assessment of the respiratory system, both in the walk test and in spirometry, showed patients in similar performance in relation to the thyroid hormonal status or the presence of PH. In fact, we expected the worst performance of GD in thyrotoxicosis and with HP. In addition, the majority of patients were oligo- or asymptomatic as to the degree of dyspnea, with 16% symptomatic among thyrotoxic and 9% among euthyroid GD patients, with 12% of GD with PH being symptomatic and 13% without PH, with no difference between groups. As for the most severe drop in SpO2, it was found in 10% of GD in both euthyroidism and thyrotoxicosis, in 8% of GD with PH, and in 13% of those without PH, also without differences. One possible explanation is based on the fact that patients with GD in thyrotoxic activity were younger and, consequently, had a greater respiratory functional reserve. On the other hand, 42 vs 48 years doesn’t seem a big difference, and might not justify the impact on the respiratory reserve. However, we didn’t find a more adequate hypothesis for this data, being a limitation of the study.

The inverse correlation between FEV1 and SPAP found in the euthyroid group, and also between the time of diagnosis and the initial SpO2 may be justified by the possibility of definitive injury to the pulmonary bed, even after normalization of thyroid hormones levels. Additionally, the inverse correlation between FT4 with FVC and FEV1 can be justified by the impairment of the hypermetabolism of the respiratory system.

In the hyperthyroid group, the inverse correlation between FT4 and % of the predicted distance and direct correlation between final heart rate and FT4 and FT3 can be attributed to the status of thyrotoxicosis. Probably, no evidence of respiratory dysfunction was observed due to the short time of diagnosis and because it was younger patients with greater respiratory reserve.

As for the limitations of our study, we have some missing data, because we were unable to apply all cardiorespiratory assessments in all patients included due to technical difficulties and follow-up of these patients, despite being a prospective study, as well as the lack of a control group. On the other hand, we have an adequate number of patients and a vast and thorough cardiorespiratory assessment of patients with Graves’ disease in a thyrotoxic and long-term state in euthyroidism, not found in the literature.

## Conclusion

In conclusion, this study reinforces the importance of screening for PH, especially with echocardiographic assessment, thus allowing early, incisive and effective treatment of patients with GD in thyrotoxicosis, both due to the high prevalence, and also the possibility of reversion and normalization of pressure pulmonary artery, avoiding irreversible complications. We also emphasize the importance of cardiorespiratory reassessment in GD, even after a long-term control of the thyrotoxic state, as we demonstrate that about 30% of these patients remain with PH and are subject to specific treatment.

Regarding the associated factors that may lead to the risk of irreversible pulmonary hypertension due to definitive changes in pulmonary circulation and vascular remodeling, we need further clarifying studies.

## Data Availability Statement

The original contributions presented in the study are included in the article/**Supplementary Material**. Further inquiries can be directed to the corresponding author.

## Ethics Statement

The studies involving human participants were reviewed and approved by the CEP UNICAMP. The patients/participants provided their written informed consent to participate in this study.

## Author Contributions

DZ-W, DO, and MP designed the research. LA collected the data. LA, DO, MP, and DZ-W analyzed and interpreted the data. LA and DZ-W wrote the manuscript. AN and MT reviewed the manuscript and provided comments. All authors contributed to the article and approved the submitted version.

## Funding

DZ-W has a research grant from the CNPq (National Council of Research) proc 302827/2018-8.

## Conflict of Interest

The authors declare that the research was conducted in the absence of any commercial or financial relationships that could be construed as a potential conflict of interest.

## Publisher’s Note

All claims expressed in this article are solely those of the authors and do not necessarily represent those of their affiliated organizations, or those of the publisher, the editors and the reviewers. Any product that may be evaluated in this article, or claim that may be made by its manufacturer, is not guaranteed or endorsed by the publisher.
