# Supplementary material for: Interplay Between Thyroid Hormone Status and Pulmonary Hypertension in Graves’ Disease: Relevance of the Assessment in Thyrotoxic and Euthyroid Patients
Source: Front Endocrinol (Lausanne). 2022 Jan 6;12:780397. doi: 10.3389/fendo.2021.780397 (PMC8772033; doi:10.3389/fendo.2021.780397)
Supplement: Supplementary file 1 [file Table_1.docx]

**SUPPLEMENTARY TABLES REGARDING THE RESPIRATORY ASESSMENT**

Table 1 - Comparative analysis between the parameters used in the respiratory asessment of patients with Graves' disease according to thyroid hormonal status.

| **RESPIRATORY ASSESSMENT** | | Euthyroidism | Hyperthyroidism | P-value |
| --- | --- | --- | --- | --- |
| Walking test risk | Low risk | 30 (73.17%) | 29 (76.32%) | 0.7481 |
|  | Medium risk | 11 (26.83%) | 9 (23.68%) |  |
| Predicted distance (m) | | 563.75 ± 69.33(574.46, 441.39-688.48) | 609.56 ± 98.1 (626.22, 452.16-781) | 0.0347 |
| Walked distance (m) | | 471.63 ± 58.02 (457, 342-597) | 475.47 ± 57.26 (478, 330-604) | 0.5140 |
| Walked distance of the predicted (%) | | 83.8 ± 13.32 (82.86, 63.47-121.08) | 78.74 ± 13.49 (79.04, 53.3-102.28) | 0.2509 |
| Drop SpO2 ≥4 points | | 4 (9.76%) | 4 (10.53%) | 1.0000 |
| Drop SpO2 | | -0.83 ± 3.19 (0, -15 – 3) | -0.45 ± 3.14 (0, -15 – 4) | 0.3340 |
| Symptomatic MRC | | 4 (9.3%) | 6 (15.79%) | 0.5032 |
| FVC or FEV1 < 80% of the predicted | | 12 (32.43%) | 10 (27.78%) | 0.6648 |
| FVC (L) | | 3 ± 0.64 (2.93, 1.94-4.63) | 3.11 ± 0.73 (3.02, 1.7-4.86) | 0.5318 |
| FVC (%) | | 89.68 ± 13.4 (89, 70-126) | 88.31 ± 15.4 (90, 56-126) | 0.9077 |
| FEV1 (L) | | 2.43 ± 0.56 (2.43, 1.68-3.68) | 2.53 ± 0.66 (2.51, 0.95-4.08) | 0.4426 |
| FEV1 (%) | | 88.49 ± 13.93 (86, 67-127) | 86.89 ± 16.27 (89, 38-116) | 0.8382 |
| FEV1/CVF | | 81.01 ± 4.88 (82.1, 68.9-94.4) | 81.51 ± 6.79 (82.05, 55.8-95.3) | 0.4141 |
| FEV1/CFV (%) | | 99.11 ± 5.89 (100, 84-113) | 98.31 ± 8 (100, 67-112) | 0.6779 |

Walking test medium risk: up to 439 meters; Walking test low risk: ≥ 440 meters; Drop SpO2: final-initial SpO2

Symptomatic MRC: Dyspnea scale of the Medical Research Council between 3 to 5; FVC: forced vital capacity; FEV1: forced expiratory volume in the first second; FEV1/FVC: forced expiratory volume in one second / forced vital capacity ratio

Categorical variables are represented by the absolute value and frequency in percentage. Continuous variables are represented by values ​​of mean ± standard deviation (median, minimum and maximum).

Table 2 - Comparative analysis between the parameters used in the respiratory assessment of patients with Graves' disease according to the presence of Pulmonary Hypertension.

| RESPIRATORY  ASSESSMENT | | PULMONARY HYPERTENSION | | | p-valor |
| --- | --- | --- | --- | --- | --- |
|  |  | Yes | No | |  |
| Walking test risk | Low | 17 (70.83%) | | 29 (76.32%) | 0.6308 |
|  | Medium | 7 (29.17%) | | 9 (23.68%) |  |
| Drop Sp02 ≥ 4 points | | 2 (8.33%) | | 5 (13.16%) | 0.6962 |
| Symptomatic MRC | | 3 (12%) | | 5 (13.51%) | 1.0000 |
| FVC or FEV1 < 80% of the predicted | | 8 (40%) | | 10 (27.03%) | 0.3146 |
| Walked distance (m) | | 472.17 ± 62.51 (457.5, 342-604) | | 481.58 ± 55.57 (483, 372-593) | 0.4100 |
| Predicted distance (m) | | 570.33 ± 104.11 (546.06, 455.62-759.84) | | 599.65±75.04 (595.62, 441.39-781) | 0.1619 |
| Predicted distance (%) | | 85.06 ± 13.86 (86.78, 53.3-112.71) | | 79.83 ±13.95 (79.22, 56.08-121.08) | 0.1103 |
| Drop SpO2 | | -1 ± 3.91 (0, -15 – 3) | | -0,53, ± 3.16 (0, -15 – 4) | 0.5556 |
| FVC (L) | | 2.87 ± 0.87 (2.72, 1.7-4.86) | | 3.13 ± 0.6 (3.07, 1.93-4.63) | 0.0712 |
| FVC (%) | | 83.7 ± 10.08 (84, 56-98) | | 91.19 ± 15.95 (91, 63-126) | 0.0993 |
| FEV1 (L) | | 2.31 ± 0.79 (2.19, 0.95-4.08) | | 2.56 ± 0.5 (2.55, 1.65-3.68) | 0.0648 |
| FEV1 (%) | | 82.05 ± 13.99 (84, 38-104) | | 90.27 ± 15.26 (90, 61-127) | 0.0543 |
| FEV1/FVC | | 79.92 ± 7.28 (81.85, 55.8-88.9) | | 82.15 ± 5.35 (82.4, 66.7-95.3) | 0.3709 |
| FEV1/FVC (%) | | 97.65 ± 9.09 (99.5, 67-108) | | 99.38 ± 6.26 (100, 80-113) | 0.7122 |

Walking test medium risk: up to 439 meters; Walking test low risk: ≥ 440 meters; Drop SpO2: final-initial SpO2

Symptomatic MRC: Dyspnea scale of the Medical Research Council between 3 to 5; FVC: forced vital capacity; FEV1: forced expiratory volume in the first second; FEV1/FVC: forced expiratory volume in one second / forced vital capacity ratio
